# Morphological and molecular characterization of *Pratylenchus* species from Yam (*Dioscorea* spp.) in West Africa

**DOI:** 10.21307/jofnem-2020-126

**Published:** 2021-01-22

**Authors:** Yao A. Kolombia, Oluwadamilola Ogundero, Emmanuel Olajide, Nicole Viaene, P. Lava Kumar, Danny L. Coyne, Wim Bert

**Affiliations:** 1 International Institute of Tropical Agriculture (IITA), PMB 5320, Oyo Road, Ibadan, Nigeria; 2 Nematology Research Unit, Department of Biology, Ghent University, K.L. Ledeganckstraat 35, 9000 Gent, Belgium; 3 Flanders Research Institute for Agriculture, Fisheries and Food (ILVO), 9820 Merelbeke, Belgium; 4 IITA, *icipe* Campus, Kasarani, P.O. Box 30772-00100, Nairobi, Kenya

**Keywords:** COI, D2-D3, Dioscorea, DNA, Ghana, Identification, Molecular, Morphology, Morphometrics, Nigeria, Phylogeny, Pratylenchus, Root-lesion nematodes, Taxonomy, West Africa, Yam

## Abstract

The root-lesion nematodes (RLN), *Pratylenchus* spp., are among the major plant-parasitic nematodes affecting yam (*Dioscorea* spp.) production in West Africa. The distribution and diversity of RLN species associated with yam was investigated through a soil and tuber survey of the main producing areas in Nigeria and Ghana. *Pratylenchus* spp. were detected in the yam rhizosphere in 59% of 81 soil samples from Ghana and 39% of 114 soil samples from Nigeria. *Pratylenchus* spp. were detected in 24 of 400 tubers examined, in combination with root-knot nematodes (*Meloidogyne* spp.) and their associated damage of galls and crazy roots (79%), and with yam nematode (*Scutellonema bradys*) and their associated damage of dry rot (17%), although no specific additional symptoms were observed for *Pratylenchus* spp. Species of *Pratylenchus* were identified by their morphological features and by sequences of the D2-D3 region of the 28 S rDNA gene and the mitochondrial cytochrome oxidase I gene (*COI*). *Pratylenchus brachyurus* was the most frequent RLN species in both the rhizosphere and tubers of yam. *Pratylenchus hexincisus* was recovered from one tuber collected in Nigeria. While further investigations are required to establish the host status of yam for this nematode, this appears to be the first record of *P. hexincisus* on yam. The present taxonomical status of *P. scribneri* and *P. hexincisus* is discussed.

Yam (*Dioscorea* spp. L.) is an economically important crop of tropical and sub-tropical areas of the world. West Africa accounts for over 93% of the total production of this tuber with Nigeria and Ghana being the main cultivating yam countries. In these countries, yam is an important staple food providing a valuable source of carbohydrates, proteins and minerals for over 380 million people from an estimated annual production of 67 MT (Nweke et al., 1991; Orkwor, 1998; Nweke, 2016; FAO, 2018). The most important yam species cultivated for food are *D. rotundata* Poir.*, D. cayenensis* Lam.*, D. alata* L.*, D. dumetorum* (Kunth) Pax.*, D. bulbifera* L. and *D. esculenta* (Lour.) Burk. Also, yam plays an important socio-cultural role among communities and its cultivation and sale serve as a major income-generating activity for the people in yam-growing areas (Onwueme and Charles, 1994). Yam production is constrained by numerous biotic factors, however, of which plant-parasitic nematodes are among the most damaging. They affect yield and tuber quality, reducing yam production and tuber storability (Ayensu and Coursey, 1972; Bridge et al., 2005; Coyne and Affokpon, 2018). The major plant-parasitic nematodes known to cause serious damage on yam tubers are the yam nematode (*Scutellonema bradys* (Steiner and LeHew, 1933; Andrássy, 1958), root-knot nematodes (*Meloidogyne* spp.) and root-lesion nematodes (RLN) (*Pratylenchus* spp.) (Bridge et al., 2005; Bridge and Starr, 2007; Kolombia et al., 2016b; Coyne and Affokpon, 2018). RLN, however, have been much less studied, even though they are known to cause dry rot symptoms in tubers, indistinguishable from the symptoms caused by *S. bradys* (Coyne et al., 2016).

*Pratylenchus coffeae* (Zimmermann, 1898) Filipjev and Schuurmans Stekhoven, 1941 is the most important RLN of yam, occurring in Central America, the Caribbean Islands and the Pacific Islands (Acosta and Ayala, 1975; Coates-Beckford and Brathwaite, 1977; Bridge, 1988; Moura and Monteiro, 1995; Bridge et al., 2005; Muniz et al., 2012; Coyne and Affokpon, 2018). In Africa, *P. brachyurus* (Godfrey, 1929) Filipjev and Schuurmans Stekhoven, 1941, *P. pseudopratensis* (Seinhorst, 1968) and *P. sudanensis* (Loof and Yassin, 1971) are known to cause damage to yam (Coyne et al., 2003; Mudiope et al., 2007; Coyne et al., 2018) with indications that they are relatively common in the yam rhizosphere and on tubers (Adegbite et al., 2008; Kolombia et al., 2020). It was also observed that *Pratylenchus* spp. were associated with the galls and crazy roots caused by root-knot nematodes, or with dry rot caused by *S. bradys*, although with no specific additional symptoms (Kolombia et al., 2016a). Being a stenomorphic genus, *Pratylenchus* is easily recognizable at the genus level (low and flattened labial region, esophageal gland lobe overlapping the intestine mostly ventrally, posterior vulva V = 70–80%, with one ovary), while morphological identification at the species level is problematic due to the low number of diagnostic features and high intraspecific variability (Luc, 1987; Duncan et al., 1999; Castillo and Vovlas, 2007). To establish the diversity of *Pratylenchus* spp., associated with yam, surveys were conducted in the main yam producing areas in Nigeria and Ghana. The *Pratylenchus* populations obtained from yam tuber tissue and yam rhizosphere were morphologically characterized and molecularly confirmed by sequencing of the D2-D3 of 28 S rDNA and mitochondrial *COI* genes.

## Materials and methods

### Nematode samples

Nematode populations used in this study were obtained soil and tuber sampling undertaken across agro-ecological zones in Ghana and Nigeria during surveys conducted between 2012 and 2015 (Table 1). Nematodes from 195 yam rhizosphere and 400 tubers were recovered using the Whitehead tray immersion technique (Hooper et al., 2005). Extraction from rhizosphere was set using 100 ml soil sub-samples including all roots retrieved from soil per sample. Tubers were peeled using a kitchen peeler, chopped and three sub-samples of 5 g tuber peels were used for the extraction (Coyne et al., 2006). Extracted nematodes were collected on 28 μm sieves, rinsed and divided: one part was heat killed and fixed in 4% formalin, the other part was fixed directly in DESS solution (Yoder et al., 2006). In total, 127 nematodes, including 75 specimens from yam tubers, were used for species identification.

**Table 1.** *Pratylenchus* spp. recovered from yam in Ghana and Nigeria, origin, code, host, altitude and GenBank numbers.

Country	Region*	District^α^	Code	Species	N	Longitude (°)	Latitude (°)	Altitude (m)	Host^T^	D2-D3	*COI*
Ghana	Brong Ahafo	Atebubu	Atebubu PS 1	*P. brachyurus*	1	0.98598	7.75467	151	*D. rotundata*^T^		
		Atebubu-Amantin	Ahontor 1	*P. brachyurus*	2	0.96798	7.79126	139	*D. rotundata*		
			Tintare 2	*P. brachyurus*	2	0.90484	7.72486	159	*D. rotundata*		
		Kintampo North	Bablioduo K 1	*P. brachyurus*	3	1.86789	8.0352	265	*D. rotundata*		
			Kintampo S 1	*P. zeae*§	6	1.84078	8.14824	206	*D. dumetorum*	MT362906, MT362907	MT952194, MT952195
	Northern	East Gonja	Adamupe 1	*P. brachyurus*	3	0.51155	8.49292	176	*D. alata*	MT362896	MT949472
			Bagabaga 1	*P. brachyurus*	2	0.61344	8.55865	157	*D. rotundata*		
			Kitoe 1	*P. brachyurus*	1	0.49596	8.4655	189	*D. rotundata*		
		Tolon	Kpalsogu 2	*P. brachyurus*	7	1.01323	9.39844	171	D. rotundata		
			Kukuo 1	*P. brachyurus*	4	1.02533	9.41156	171	*D. rotundata*		
			Kukuo 2	*P. brachyurus*	2	1.02556	9.41114	170	*D. rotundata*		
			Kukuo 3	*P. brachyurus*	2	1.02556	9.41114	170	*D. rotundata*		
			Wala 1	*P. brachyurus*	2	1.24929	9.63993	124	*D. rotundata*		
Country	State*	LGA^α^	Code	Species	N	Longitude (°)	Latitude (°)	Altitude (m)	Host^T^	D2-D3	*COI*
Nigeria	Abia	Umuahia	Umudike 1	*P. brachyurus*	1	7.53057	5.48212	108	*D. rotundata*		
			Umudike 2	*P. brachyurus*	2	7.53057	5.48212	108	*D. rotundata*		
		Umuahia North	Umuagu 1	*P. zeae*	1	7.44739	5.61234	90	*D. dumetorum*		
	Anambra	Anambra East	Igbariam 1	*P. brachyurus*	1	6.96508	6.30112	69	*D. rotundata*^T^		
	Benue	Otukpo	Otukpo 1	*P. hexincisus*	12	8.13327	7.19212	196	*D. alata*^T^	MT362904, KY828292	MT951588, KY828320
	Ekiti	Irepodum-Ifelodum	Araromi 1	*P. brachyurus*	2	5.19352	7.67682	450	*D. rotundata*^T^		
	Enugu	Udi	Amoka 1	*P. brachyurus*	7	7.39581	6.55556	388	*D. cayenensis*		
	Imo	Owerri	Mbaise 1	*P. brachyurus*	14	7.03433	5.48433	233	*D. rotundata*^T^	MT362898, MT362899, MT362900, MT362901	MT949474, MT949475
	Kogi	Idah	Ega 1	*P. brachyurus*	1	6.72912	7.10123	29	*D. rotundata*^T^		
			Ega 2	*P. brachyurus*	3	6.72912	7.10123	29	*D. rotundata*^T^		
			Ega 3	*P. brachyurus*	1	6.72912	7.10123	29	*D. rotundata*^T^		
		Ijumu	Okejumu 1	*P. brachyurus*	5	5.93338	7.84627	495	*D. rotundata*^T^		
	Nasarawa	Lafia	Rimiuka 1	*P. brachyurus*	21	8.51598	8.49365	175	*D. rotundata*		
			Rimiuka 2	*P. brachyurus*	6	8.51598	8.49365	175	*D. rotundata*^T^		
		Nasarawa Eggon	Eggon 1	*P. brachyurus*	8	8.5409	8.71445	271	*D. rotundata*^T^	MT362897	

§ Kintampo S1: Two species were recorded from the same sample *P. brachyurus* (*n* = 3) and *P. zeae* (*n* = 6); ^T^:Sample from yam tuber, otherwise, sample are from rhizosphere. ^*^State (Nigeria)/Region (Ghana); ^α^:LGA = Local Government Area (Nigeria)/District (Ghana); ^T^:Sample from yam tuber, otherwise, sample are from rhizosphere. ^§^:Kintampo S 1: Two species were recorded from the same sample *P. brachyurus* (n = 3) and *P. zeae* (n = 6).

### Morphological characterization

Nematodes from 27 samples fixed in formalin were processed to anhydrous glycerin following the glycerin-ethanol method (Seinhorst, 1959) as modified by De Grisse (1969). Permanent slides were prepared and used to record morphometrics and morphological features (Castillo and Vovlas, 2007; Inserra et al., 2007) using an Olympus BX51 DIC microscope equipped with a Nikon digital camera. Additional morphological and morphometrical data were recorded from temporary slides made from DESS fixed specimens, before DNA extraction (see Table 1).

### Molecular characterization

Following morphological identification, the same individual nematodes were picked from temporary slides and used for extraction of genomic DNA using a quick alkaline lysis protocol (Janssen et al., 2016). DNA was amplified by preparing 24 μl PCR master mix comprising 16 μl double sterilized distilled water, 2.5 μl 10x buffers, 2 μl MgCl2, 0.05 μl of dNTP (10 mM), 1 μl of reverse and forward primers, 0.05 μl of Toptaq and 2 μl of nematode template DNA. The primer set D2A (5’–ACA AGT ACC GTG AGG GAA AGT TG–3’) and D3B (5’–TCG GAA GGA ACC AGC TAC TA–3’) (Subbotin et al., 2006) was used for amplification of the D2-D3 expansion regions of 28 S rDNA gene and the cytochrome c oxidase subunit 1 (*COI*) gene fragment was amplified using the primer set JB3Prat (5’–TTT TTT GGG CAT CCT GAA GTC TAT–3’) and JB4Prat (5’–CCT ATT CTT AAA ACA TAA TGA AAA TG–3’) following DNA amplification profile described in Kolombia (2017).

PCR products were electrophoretically separated on a 1% agarose gel and stained with ethidium bromide. PCR products were purified using the Wizard® SV Gel and PCR Clean-Up System Kit (Promega, the Netherlands) as described in the manufacturer’s instructions and sequenced by Macrogen Inc. (the Netherlands) in forward and reverse directions. Consensus sequences were assembled using GENEIOUS 9.15 (Biomatters; http://www.geneious.com) and deposited in the NCBI GenBank (Table 1).

### Phylogenetic analysis

Both D2-D3 of 28 S rDNA and *COI* of mtDNA sequence datasets were aligned using MUSCLE (Edgar, 2004) with default settings. Outgroup taxa of each dataset were chosen based on previously published data (Subbotin et al., 2008; Liu et al., 2016). The best fit models of DNA evolution were estimated using the program jModeltest 0.1.1 (Posada, 2008) under the Akaike information criterion (AIC). Bayesian phylogenetic analysis (BI) was undertaken using MrBayes 3.2.6 for 1 × 10^6^ generations with a general time-reversible model with a gamma distribution for the remaining sites (GTR + I + G), four runs, 20% burn-in, and subsampling frequency of 500 generations (Huelsenbeck and Ronquist, 2001) for both D2-D3 and *COI*.

## Results

### Occurrence and morphological characterization of *Pratylenchus* spp. from yam

**Figure 1:**
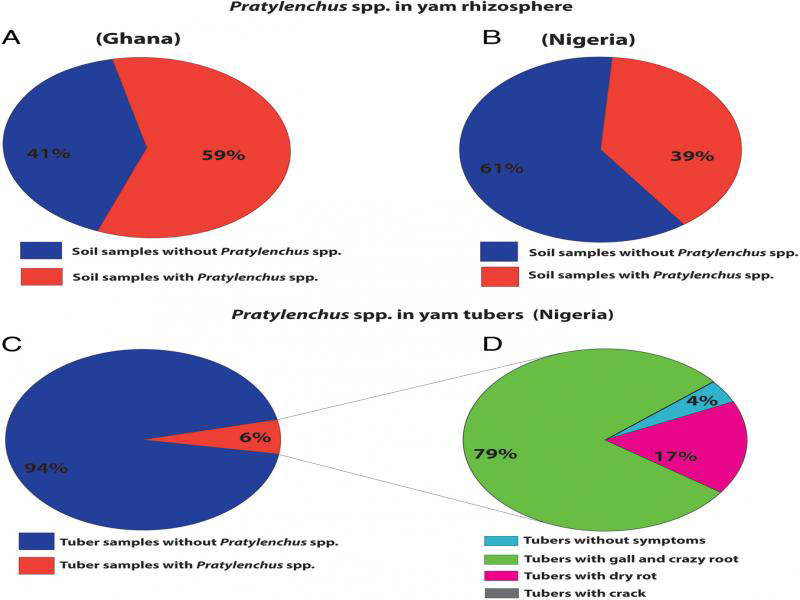
Proportion of *Pratylenchus* spp. in the yam rhizosphere from Ghana “*n* = 81” (A) and Nigeria “*n* = 114” (B), in yam tubers “*n* = 400” (C) and of nematode damage symptoms on yam tubers (D)

From the rhizosphere, *Pratylenchus* spp. were detected in 48 samples (59%) collected in Ghana (Fig. 1A) and 45 samples (39%) in Nigeria (Fig. 1B). The density of *Pratylenchus* spp. from the rhizosphere varied from 2 to 704 individuals per 100 ml soil and roots in Ghana, and from 2 to 398 individuals in 100 ml of soil and roots in Nigeria. From 400 tubers examined, *Pratylenchus* spp. were recovered from just 6% of the 400 tuber peels (Figure 1C). Twenty-four tubers were infected with *Pratylenchus* spp., of which, 19 tubers (79%) also had galling and crazy root damage caused by the root-knot nematode (*Meloidogyne* spp.), 4 tubers (17%) showed dry rot symptoms caused by the yam nematode (*Scutellonema bradys*) while no symptoms were observed in one tuber, which had a density of 50 specimens of *Pratylenchus brachyurus*, per 5 g of yam peels (Figure 1D). Densities of *Pratylenchus* spp. were as higher as 340 nematodes in tubers with symptoms and up to 525 individuals per 5 g of yam peels in tubers with dry rot and galling, respectively. Twenty-eight populations from 12 yam tubers and 16 rhizosphere samples were studied using morphological and molecular data, which resulted in the identification of *Pratylenchus brachyurus* and *P. hexincisus* (Taylor and Jenkins, 1957) and *P. zeae* (Graham, 1951).

*Pratylenchus brachyurus* was the most prevalent RLN species in Ghana and Nigeria, present in 11 of the 12 tubers used for species identification and 88% of *Pratylenchus*-positive rhizosphere samples. Twenty-five specimens per 5 g of yam peels of *Pratylenchus hexincisus* were recovered in just one tuber showing galls from Nigeria, and *P. zeae* was detected in 12% of the rhizosphere samples from Ghana (26 nem/100 ml soil) and Nigeria (3 nem/100 ml soil).

#### Systematics

**Figure 2:**
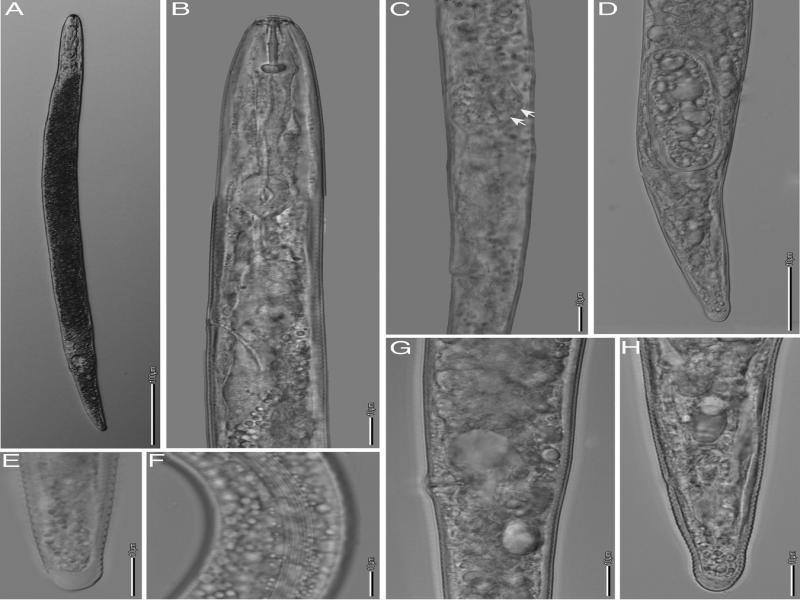
*Pratylenchus brachyurus*. Light micrographs of Female: A: Entire body; B: Esophageal region; C: Spermatheca with sperm cells; D: Posterior end of gravid female; E: Tail end; F: Lateral field at mid body; G: Vulva; H: Tail; (scale bars: B-H = 10 μm; A = 100 μm).

**Figure 3:**
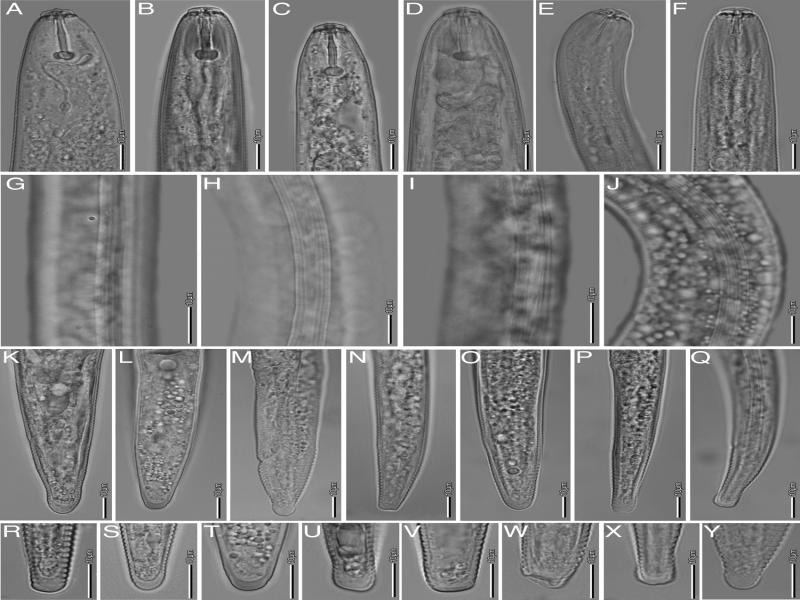
Morphological variations in *Pratylenchus brachyurus*. A-F: Anterior regions (A-F); Lateral field at mid body (G-J); Tail region (K-Q); and Tail end (R-Y); (scale bars: 10 μm).

*****Pratylenchus brachyurus***[Bibr ref035][Bibr ref033]** (Figures 2 and 3; Tables 2 and 3).

**Table 2.** Measurements of thirteen *Pratylenchus brachyurus* populations from Ghana. All measurements are in μm and in the form: mean ± s.d. (range).

Sample	Atebubu PS 1	Ahontor 1	Tintare 2	Bablioduo K 1	Kintampo S 1	Kpalsogu 2	Kukuo 1
N	1♀*	2♀♀	2♀♀	3♀♀	3♀♀	7♀♀♀*	4♀♀
L	507	566-543	518-532	556±16.9 (538-572)	487±44.7 (451-537)	470±38 (390-504)	501±13.6 (488-520)
a	16.5	24.0-21.0	18.9-21.9	24.4±5.4 (19.6-30.2)	18.2±0.72 (17.4-18.7)	16.7±2.3 (14.1-21.1)	19.9±3.3 (15.8-22.6)
b	6.2	4.6-4.6	5.3-5.7	5.8±0.64 (5.3-6.2)	5.2±0.9 (4.2-5.8)	4.7±0.21 (4.5-4.8)	5.2±1.2 (4.2-6.9)
b'	4.3	3.8-4.1	4.2-4.7	4.1±1.0 (3.4-5.3)	3.7±0.74 (3.1-4.5)	3.5±0.07 (3.4-3.5)	4.1±0.62 (3.4-4.8)
C	21.6	17.1-17.2	20.6-17.6	24.7±4.3 (19.9-28.3)	16.4±1.1 (15.3-17.5)	16.7±2.0 (14.5-19.5)	17.4±1.3 (15.7-18.7)
c'	1.5	2.8-1.7	1.6-2.0	1.6±0 (1.6-1.6)	1.9±0.21 (1.7-2.1)	1.8±0.33 (1.4-2.3)	1.8±0.27 (1.6-2.2)
V%	84.8	86.0-83.0	82.0-83.0	85.9±0.17 (85.7-86.0)	85.7±0.58 (85.0-86.0)	83.5±3.3 (77.0-88.0)	84.3±1.5 (82-85)
Stylet length	19.1	19.6-18.8	18.1-18.2	19.2±0.2 (19.0-19.4)	18.3±0.12 (18.2-18.4)	17.1±0.64 (16.6-18.4)	19.1±0.68 (18.5-20.1)
Stylet knob width	5.7	5.2-4.1	5.3-5.7	4.4±0 (4.4-4.4)	4.9±0.9 (4.0-5.8)	5.2±0.7 (4.3-6.3)	5.2±0.51 (4.6-5.8)
Stylet knob height	3.8	3.1-3.0	3.9-3.3	2.9±0.57 (2.5-3.3)	3.0±0.65 (2.3-3.6)	3.6±0.54 (2.8-4.2)	3.5±0.35 (3.1-3.8)
DEGO from stylet base	3.7	2.8-3.3	2.6-3.1	3.6±0.51 (3.2-4.2)	3.8±0.64 (3.1-4.2)	2.4±0.57 (2.0-2.8)	2.7±0.4 (2.3-3.1)
Anterior end to:							
centre of metacorpus	53.5	72.4-62.5	50.8-48.2	56.7±4.9 (52.4-62.1)	52.7±4.6 (49.2-57.9)	51.0±6.1 (42.9-61.7)	54.9±6.4 (47.6-63.1)
median bulb base	61.5	80.7-69.2	59.2-55.8	63.6±3.6 (61.2-67.8)	61.4±4.4 (57.8-66.3)	57.2±7.2 (46.3-69.2)	61.1±6.9 (53.4-70)
Cardia	81.1	123-117	97.1-93.7	98.4±9.1 (92.0-105)	94.6±12.4 (83.2-108)	103±9.4 (96.8-110)	101±21.6 (72.6-124)
end of esophageal gland end	117	147-134	125-113	140±28.1 (108-160)	135±17.5 (119-154)	139±5.1 (135-142)	126±22.0 (103-154)
secretory/excretory pore	71.3	111-87.5	71.6-79.4	91.7±8.2 (85.4-101)	72.4±3.1 (70.2-74.6)	72.1±9.0 (56.9-79.8)	85.5±4.1 (81.6-89.8)
Esophagus overlap	51.1	21.6-18.3	27.7-18.9	38.3±10.0 (31.2-45.3)	37.8±6.3 (31.6-44.2)	39.7±0.64 (39.2-40.1)	29.6±8.3 (24.1-41.7)
Max. body diam.	30.7	23.6-25.8	27.4-24.2	23.4±4.7 (18.9-28.3)	26.7±1.9 (25.3-28.8)	28.5±3.1 (23.8-34.2)	25.7±4.4 (22.0-30.9)
Vulval body diam.	24.2	19.0-21.4	22.6-20.6	21.7±3.8 (18.6-25.9)	21.4±1.2 (20.2-22.5)	22.6±2.7 (20.4-27.3)	21.4±1.8 (19.3-23.7)
Anal body diam.	15.6	11.8-18.8	16.2-14.8	14.6±3.0 (12.4-18.0)	15.6±0.61 (15.1-16.3)	15.5±2.1 (11.8-18)	16.1±1.6 (14.5-18.1)
Anterior genital		178-169	167-	–	186±79.8 (126-277)	124±11.1 (116-132)	86.1±23.1 (71.8-121)
Spermatheca-vagina		-35.7	43.3-	–	63.9±28.4 (45.9-96.6)	37.4±9.1 (30.9-43.8)	32±11.4 (24.6-45.2)
Tail length	23.5	33.1-31.6	25.2-30.1	23.0±4.3 (20.2-28.0)	29.8±4.5 (27-35)	28.4±3.3 (24.4-32.6)	28.9±2.4 (26-31.7)
Number of tail annuli	15	19.0-18.0	15.0-19.0	18.5±2.1 (17-20)	18.0±2 (16.0-20.0)	18.0±0.82 (17.0-19.0)	17.3±1.7 (15-19)
Vulva to anus distance	49.7	57.0-57.5	64.4-58.7	–	42.5±6.0 (37.7-49.2)	45.9±7.6 (39.4-58.9)	51.1±3.3 (47.5-55.4)
Post-uterine sac	33.2	20.1-22.6	24.7-24.9	–	22.3±4.2 (17.6-25.7)	21.2±4.4 (16.5-25.1)	17.1±2.8 (13.6-19.5)
Lateral field width	–	11.3-11.9	10.7-10.3	–	–	–	7.4±0.51 (6.7-7.9)
Sample		Kukuo 2	Kukuo 3	Wala 1	Bagabaga 1	Adamupe 1	Kitoe 1
N		4♀♀	2♀♀	2♀♀	2♀♀	3♀♀*	1♀*
L		541±46.1 (501-607)	488-541	516-560	522-516	549±60.1 (480-590)	549
a		20.3±2.6 (18.7-24.2)	20.3-	23.5-23.7	18.1-19.1	18.9±4.6 (15.4-24.1)	16.0
b		5.3±0.29 (4.9-5.6)	–	5.0-4.8	5.1-5.3	4.9±1.8 (2.9-6.3)	5.6
b'		3.8±0.29 (3.5-4.1)	–	3.9-3.9	4.0-4.2	4.1±1.5 (2.5-5.4)	4.1
c		17.1±2.4 (14.3-20)	16.7-19.7	17.3-16.4	18.7-20.5	17.0±1.7 (15.8-19.0)	25.1
c'		2.0±0.19 (1.7-2.1)	1.8-1.6	2.5-2.3	1.7-1.5	1.9±0.36 (1.6-2.3)	1.5
V%		84.5±1 (83.0-85.0)	85.0-84.0	85.0-83.0	85.0-83.0	85.0±1.2 (84.2-86.4)	85.7
Stylet length		18.7±0.21 (18.4-18.9)	19.3-18.2	19.4-20.0	19.3-19.8	18.9±0.49 (18.3-19.2)	19.8
Stylet knob width		5.6±0.7 (5-6.6)	4.3-5.2	5.2-4.3	6.1-6.0	5.6±0.25 (5.3-5.8)	4.9
Stylet knob height		3.6±0.22 (3.4-3.9)	3.7-3.9	3.2-3.2	3.7-3.7	3.5±0.46 (3.2-4)	3.7
DEGO from stylet base		3.1±0.25 (2.8-3.4)	–	4.0-3.2	4.3-2.9	2.7±0.55 (2.1-3.1)	3.0
Anterior end to:							
centre of metacorpus		58.1±4.8 (53.2-62.6)	52.1-55.7	62.4-61.6	55.6-53.0	62.5±0.98 (61.4-63.3)	61.2
median bulb base		65.9±4.8 (61.6-71.2)	57.2-61.4	71.3-72.1	64.8-61.2	70.6±0.57 (70.1-71.2)	67.3
Cardia		102±7.0 (92.5-108)	–	104-116	102-96.7	121±37.6 (91.3-164)	97.6
end of esophageal gland end		142±9.3 (129-150)	–	133-144	130-122	144±42.5 (108-191)	133
secretory/excretory pore		87.6±7.5 (78.1-96.3)	–	99.9-89.9	81.0-70.6	90.5±10.7 (79.3-101)	
Esophagus overlap		36.2±3.8 (31.1-39.5)	–	35.9-38.4	39.0-29.9	25.3±9.4 (18.0-35.9)	38.0
Max. body diam.		27.1±4.9 (20.7-32.4)	24.0-	21.9-23.6	28.8-27	29.7±4.7 (24.5-33.5)	34.2
Vulval body diam.		21.9±2.6 (18.5-24.9)	20.1-22.3	18.1-20.7	22.2-22.8	24.4±3.0 (21.0-26.4)	20.6
Anal body diam.		16.4±1.2 (14.8-17.5)	16.2-17.6	12.0-15.1	16.6-16.8	17.3±1.2 (16.4-18.6)	14.9
Anterior genital		180±42.2 (133-234)	–	–	219-210	193±90.6 (129-257)	
Spermatheca-vagina		49.4±14.3 (37.9-65.4)	–	–	38.3-42.0	–	
Tail length		31.9±3.1 (29.8-36.5)	29.2-27.4	29.8-34	27.9-25.2	32.5±4.3 (29.6-37.4)	21.9
Number of tail annuli		18.5±2.4 (16.0-21.0)	16.0-15.0	18.0-16.0	17.0-16.0	21.7±3.2 (18.0-24.0)	
Vulva to anus distance		52.8±6.7 (43.0-58.2)	44.4-51.7	48.1-59.1	54.3-55.7	49.9±9.7 (42.8-61)	51.5
Post-uterine sac		21.1±1.9 (19.7-23.9)	–	18.1-19.2	31.5-23.9	18.9±1.7 (17.2-20.5)	49.5
Lateral field width		10.2±0.71 (9.6-11.0)	–	–	11.8-10.6	11.5±1.8 (10.2-12.7)	

* Morphometrics derived from temporary slides; otherwise, morphometrics derived from permanent slides.

**Table 3.** Measurements of thirteen *Pratylenchus brachyurus* populations from Nigeria.

Sample	Igbariam 1	Araromi 1	Okejumu 1	Ega 1	Ega 2	Ega 3	Rimiuka 2
N	1♀	2♀♀	5♀♀	1♀	3♀♀	1♀	6♀♀
L	461	563–584	493±55.9 (435–563)	470	472±33.9 (445–510)	536	545±30.2 (507–579)
a	15.7	22.6–19.3	16.6±0.72 (15.7–17.4)	16.6	18.0±3.1 (15.0–21.2)	21.0	19.8±1.5 (18.6–22.7)
b		–	–		–		–
b’	3.8	3.9–3.9	3.7±0.59 (2.9–4.2)	3.3	–	5.0	4.2±0.22 (3.9–4.4)
C	21.8	20.1–17.3	19.8±4.7 (15.0–26.6)	22.0	19.6±6.1 (14.3–26.3)	21.7	24.1±5.6 (19.6–34.9)
c’	1.5	1.7–1.9	1.5±0.34 (0.93–1.8)	1.6	1.9±0.47 (1.5–2.4)	1.7	1.6±0.21 (1.2–1.8)
V%	85.0	87.0–85.0	85.6±1.1 (84.0–87.0)	85.0	84.7±0.58 (84–85)	86.0	84.7±1.2 (83–86)
Stylet length	17.1	20.4–20.4	18.9±0.63 (18.0–19.6)	19.4	19.3±0.64 (18.6–19.7)	19.1	18.6±0.44 (18.1–19.1)
Stylet knob width	4.2	5.8–5.8	5.0±0.78 (3.8–5.9)	4.7	4.9±0.51 (4.3–5.3)		5.2±0.54 (4.4–5.8)
Stylet knob height	3.4	3.8–4.0	3.1±0.51 (2.5–3.7)	2.9	3.1±0.0 (3.1–3.1)		3.3±0.21 (3.1–3.6)
DEGO from stylet base		2.8–2.9	2.9±0.24 (2.6–3.1)	3.3	–		2.7±0.99 (2–3.4)
*Anterior end to:*							
centre of metacorpus	52.4	61.5–64.2	61.9±11.1 (47.3–74.1)	58.0	52.2±3.6 (48.4–55.6)	54.6	58.8±7.3 (51.2–69.3)
median bulb base	58.2	71.3–72.7	69.9±10.6 (56.7–81.8)	66.8	59.1±3.6 (55.6–62.8)	62.0	66.4±6.7 (58–75.6)
cardia		–	–		–		–
end of esophageal gland end	120	146–151	135±18.4 (110–150)	144	–	107	132±8.6 (123–143)
secretory/excretory pore	63.9	97.0–98.1	89.3±12.1 (77.3–110)	87.5	79.6±5.9 (75.4–83.8)	81.3	90.6±10.0 (84.1–111)
Esophagus overlap	21.1	35.5–38.6	36.4±4.8 (32.9–43.2)	31.1	–	21.8	28.9±4.3 (23–32.9)
Max. body diam.	29.3	24.9–30.2	28.7±1.6 (27.4–30.9)	28.3	26.6±4.3 (21.7–29.6)	25.5	27.7±1.5 (25.5–29.2)
Vulval body diam.	21.5	22.1–24.1	23.4±1.5 (21–24.7)	21.3	20.8±3.7 (16.6–23.5)	20.6	22.9±1.2 (21.3–24.6)
Anal body diam.	13.7	16.6–17.8	18.0±2.9 (15.6–22.8)	13.5	13.6±2.5 (11.3–16.3)	14.8	14.7±0.9 (13.4–15.8)
Anterior genital		–	–		–		–
Spermatheca-vagina		–	–		–		–
Tail length	21.1	28.0–33.8	25.6±4.3 (21.2–32.2)	21.3	25.8±7.9 (16.9–32.2)	24.7	23.4±3.7 (16.1–25.9)
Number of tail annuli		–	–		–		–
Vulva to anus distance	42.7	47.0–53.8	44.3±6.5 (39.2–53.5)	49.2	48.8±9.8 (37.6–55.9)	47.4	59.1±8.6 (49.0–72.7)
Post-uterine sac		20.9–20.2	18.0±1.6 (16.4–19.9)	19.9	–		16.6±2.8 (14.6–18.6)
Lateral field width		–	–		–		–
Sample	Eggon 1		Umudike 1	Umudike 2	Amoka 1	Rimiuka 1	Mbaise 1
N	4♀♀*	4♀♀	1♀	2♀♀	7♀♀	21♀♀	14♀♀
L	568 ± 57 (515–648)	578±56.1 (519–648)	486	551–599	591±42.5 (556–679)	509±84.1 (394–641)	569±31.4 (510–613)
a	20.7±3.8 (18.6–26.3)	18.7±1.4 (17.4–20.6)	15.6	23.4–24.0	21.7±2.5 (18.3–24.7)	20.3±2.9 (16.0–24.8)	22.6±2.8 (17.0–27.0)
b	–	–	3.0	4.7–4.8	5.0±0.6 (4.3–5.8)	–	7.5±0.93 (6.3–8.8)
b’	–	4.8±0.57 (4.1–5.5)	2.5	4.4–4.3	4.0±0.48 (3.3–4.5)	3.5±0.52 (2.7–4.5)	4.4±0.59 (3.6–5.5)
C	25.1±9.3 (19.5–39)	23.6±2.2 (21.3–25.5)	16.4	26.2–20.6	20.4±6.3 (15.8–34.1)	18.7±3.0 (13.4–24.4)	19.8±1.6 (17.9–23.2)
c’	1.6±0.33 (1.2–2.0)	1.5±0.13 (1.3–1.6)	1.8	1.3–1.8	1.9±0.21 (1.6–2.3)	1.7±0.18 (1.5–2.1)	2.0±0.37 (1.5–2.5)
V%	85.5±0.58 (85.0–86.0)	85.0±0.82 (84.0–86.0)	83.6	86.0–84.0	85.6±0.53 (85.0–86.0)	84.9±1.7 (81.0–87.0)	84.6±1 (82–86)
Stylet length	17.8±1.0 (16.3–18.4)	18.8±0.57 (18.3–19.6)	19.1	20.5–18.5	19.1±0.6 (18.3–20.1)	19.4±1.0 (17.2–20.9)	18.9±0.69 (17.8–19.8)
Stylet knob width	5.3±0.49 (4.7–5.6)	4.8±0.46 (4.1–5.1)	5.6	5.1–5.5	5.6±0.28 (5.0–5.8)	5.0±0.46 (4.1–6.0)	5.1±0.52 (4.3–5.7)
Stylet knob height	3.4±0.21 (3.2–3.6)	3.6±0.18 (3.4–3.8)	4.0	3.4–3.4	3.9±0.45 (3.2–4.5)	3.0±0.52 (2.0–3.7)	3.5±0.46 (2.8–4.6)
DEGO from stylet base	3.6±0.49 (3.2–3.9)	3.0±0.45 (2.6–3.6)	2.1	2.4–3.3	3.2±0.56 (2.4–3.9)	3.2±0.51 (2.4–4.2)	3.0±0.47 (2.3–3.7)
*Anterior end to:*							
centre of metacorpus	58.0±7.1 (50.6–67.0)	59.4±5.3 (55.7–67)	63.3	58.9–61.3	62.9±1.5 (60.0–65.2)	64.0±4.7 (54–72.6)	61.7±5.1 (55.5–72.9)
median bulb base	66.0±8.0 (57.4–76.5)	67.8±5.9 (63.2–75.9)	71.2	66.2–69.0	70.7±1.7 (68.7–73.8)	72.4±4.2 (62.2–78.4)	70.5±4.7 (65.1–81.3)
Cardia	–	–	164	118–125	120±9.5 (104–131)	–	78.2±9.1 (68.9–91.3)
end of esophageal gland end	–	122±4.7 (117–127)	191	127–139	149±11.4 (134–167)	148±17.1 (113–181)	130±15.3 (103–149)
secretory/excretory pore	102±3.5 (98.8–106)	84.4±13.7 (74.2–100)	91.5	87.1–98.9	96.4±5.5 (89.3–102)	96.9±10.3 (82.8–128)	93.6±11.6 (76.5–114)
Esophagus overlap	29.2±1.9 (27.8–30.5)	27.5±2.5 (24.1–29.5)	35.9	40.9–18.8	26.8±7.8 (17.6–39.6)	37.9±9.5 (12.6–50.6)	30.0±8.7 (14.6–45.9)
Max. body diam.	27.8±2.5 (24.6–30.1)	31.0±2.5 (28.3–34.2)	31.1	23.5–24.9	27.5±2.6 (24.5–31.2)	25.3±3.5 (21.6–33.4)	25.7±4.3 (21.8–34.2)
Vulval body diam.	23.4±1.6 (21.4–25.2)	24.2±1.6 (22.4–26)	25.9	20.2–19.4	23.1±2.3 (20.3–26.3)	22.1±2.8 (17.4–28.0)	20.7±3.5 (16.6–28.2)
Anal body diam.	15.9±3.4 (11.4–18.6)	16.7±1.2 (15.8–18.4)	16.9	16.0–16.6	16.0±3.2 (10.0–19.5)	16.0±1.8 (12.6–18.7)	14.8±2.6 (11.2–19.3)
Anterior genital	–	–	129	141–198	166±66.1 (84.3–243)	–	206±43.6 (162–261)
Spermatheca-vagina	–	–	24.2	37.5–39.4	37.5±16.2 (20.1–55.0)	–	51.1±25.7 (17.1–77.6)
Tail length	24.7±7.7 (13.2–29.7)	24.5±2.2 (21.6–26.7)	29.6	21.0–29.1	30.6±6.9 (17.7–37.4)	27.3±2.2 (23.1–32)	28.8±2.5 (25–32.1)
Number of tail annuli	–	–	18.0	16.0–	21.9±1.5 (20.0–24.0)	–	23.8±2.2 (21–27)
Vulva to anus distance	60.0±7.8 (50.3–66.9)	57.7±8.8 (51.2–70.7)	45.9	60.9–65.7	52.9±11.4 (42.8–73.2)	48.5±9.1 (25.9–62.9)	58.9±3.7 (54.2–66.7)
Post-uterine sac	–	19.5±2.9 (16.2–21.5)	19.1	12.3–14.2	19.9±3.0 (17.2–25.1)	18.9±4.7 (14.3–34.9)	19.8±1.8 (16.1–22.8)
Lateral field width	–	–	12.7	8.4–7.7	9.4±1.6 (7–10.8)	–	7.3±1.1 (6.1–8.1)

* Morphometrics derived from temporary slides; otherwise, morphometrics derived from permanent slides. All measurements are in μm and in the form: mean±s.d. (range)

Female:

Body small 390–679 μm long, stout to moderately slender. Habitus almost straight when heat-relaxed. Lateral fields usually with four longitudinal lines; sometimes 4 to 6 lateral lines at mid body or 2 additional lateral fields faint or broken. Cephalic region slightly offset from body, with two lip annuli. Robust stylet (16.3–20.9 μm long) with stout and rounded basal knobs, 3.8–6.6 μm wide, with irregular shape on the surface. The dorsal esophageal gland opening (DEGO) at 2.0–4.3 μm posterior the stylet base. Median bulb muscular, rounded to oval. Excretory pore just anterior to region of esophago-intestinal junction, but often indistinct. Esophageal glands overlapping intestine ventrally and sometimes laterally. Reproductive system monodelphic-prodelphic, ovary with oocytes in one row, occasionally two rows. Spermatheca usually indistinct, if present, well developed, rounded to spherical, filled with sperm cells in a few specimens. Vulva at 77–88% of body length. Post-vulval uterine sac generally shorter than body diameter length (12.3–34.9 μm long). Vulva-anus distance about twice the tail length. Tail slightly tapering, terminus mostly bluntly rounded, varying from somewhat narrower, flat to slightly indented; terminus smooth.

*Males*: Not observed.

*P. brachyurus* populations described were collected from yam tubers and rhizosphere from five districts in Ghana and ten Local Government Areas (LGA) in Nigeria.

From the morphology and the morphometrics, the studied populations are in agreement with the original description of *P. brachyurus*, and to subsequent descriptions (Roman and Hirschmann, 1969; Corbett, 1976; Castillo and Vovlas, 2007). However, the spermatheca was filled with sperm cells in two specimens (of the same sample), which has not previously been observed. In addition, in one specimen, the vulva was located at 77% of the body, while the vulva is normally located at 81–88% of the body.

**Figure 4:**
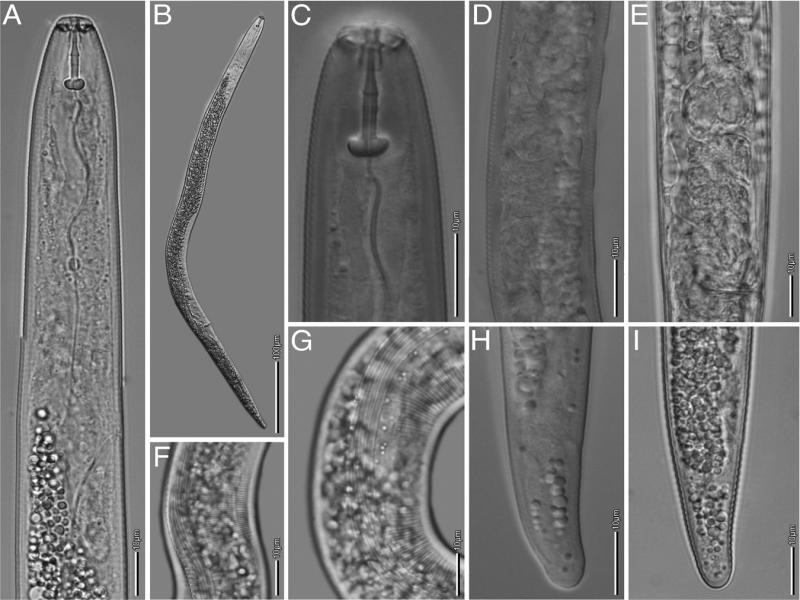
Light micrographs of female *Pratylenchus hexincisus*. A: Anterior end; B: Entire body; C: Head; D-E: Reproductive track; F-G: Lateral field at mid body; H-I: Tail; (scale bars: A, C-I = 10 μm; B = 100 μm).

**Pratylenchus hexincisus** Taylor and Jenkins, 1957. (Figure 4 and Table 4).

**Table 4.** Measurements of a *Pratylenchus hexincisus* population from Nigeria.

Sample	Otukpo 1	
n	5♀♀*	7♀♀
L	503±99 (367–625)	427±34.7 (382–492)
a	18.4±3.1 (16.4–22)	22.4±1.4 (20.8–24.6)
b	–	6.1±0.52 (5.6–7)
b’	–	4.4±0.71 (3.8–5.6)
c	15.9±3.5 (13.5–19.9)	12.8±1.9 (10.8–15.7)
c’	2.4±0.31 (2.1–2.7)	2.6±0.42 (2.2–3.4)
V%	75.7±1.6 (74.1–78)	74.9±1.8 (72.6–78)
Stylet length	13.9±0.43 (13.6–14.5)	14.9±1.6 (11.8–16.1)
Stylet knob width	4.3±0.27 (3.9–4.5)	2.1±0.19 (2.0–2.4)
Stylet knob height	2.6±0.28 (2.3–2.9)	2.4±0.21 (2.2–2.6)
DEGO from stylet base	3.3±0.34 (2.8–3.6)	4.4±0.87 (3.8–5.4)
*Anterior end to:*		
centre of metacorpus	50.0±4.1 (46.8–57.0)	45.5±5.8 (36.0–50.7)
median bulb base	57.4±3.6 (54.4–63.5)	54.3±4.3 (48.5–58.8)
cardia	–	70.6±7.5 (59.2–81.1)
end of esophageal gland end	–	101±17.2 (74.4–124)
secretory/excretory pore	–	54.7±3.9 (50.0–59.2)
Esophagus overlap	–	–
Max. body diam.	24.3±2.2 (22.3–26.6)	19.2±1.7 (17.4–22.4)
Vulval body diam.	–	20.9±4.8 (17.4–30.3)
Anal body diam.	14.6±0.51 (14–15.2)	13.1±1.8 (11.0–15.2)
Anterior genital	–	93.7±17.5 (81.3–106)
Spermatheca-vagina	–	–
Tail length	36.1±4.1 (31.4–39.0)	34.0±4.7 (28.8–39.7)
Number of tail annuli	–	24.0±2.8 (22.0–26.0)
Vulva to anus distance	–	–
Post-uterine sac	–	–
Lateral field width	–	7.0±0.28 (6.8–7.2)

* Morphometrics derived from temporary slides; otherwise, morphometrics derived from permanent slides. All measurements are in μm and in the form: mean ± s.d. (range).

Female:

Body small, 367–625 μm long, stout to moderately slender. Habitus slightly straight when heat-relaxed. Lateral fields indistinct; when observed, with four to six longitudinal lines at mid body. Lateral field 6.8–7.2 μm wide at mid body with crenated margins (Figure 4). Short stylet 15 µm (11.8–16.1 μm), with rounded knobs. Median bulb oval. Cephalic region slightly offset from body, with two annuli. Esophageal glands overlapping intestine ventrally and laterally. Spermatheca rounded and obscure. Vulva located at 72.6–78%. Tail slightly tapering, terminus mostly broadly rounded.

*Males*: Not observed.

*Remarks:* The population used in this study is from one location (Otukpo) in Nigeria collected from a yam tuber.

The studied population was in agreement with the original description of *P. hexincisus* and to subsequent descriptions (Castillo and Vovlas, 2007; Inserra et al., 2007).

**Figure 5:**
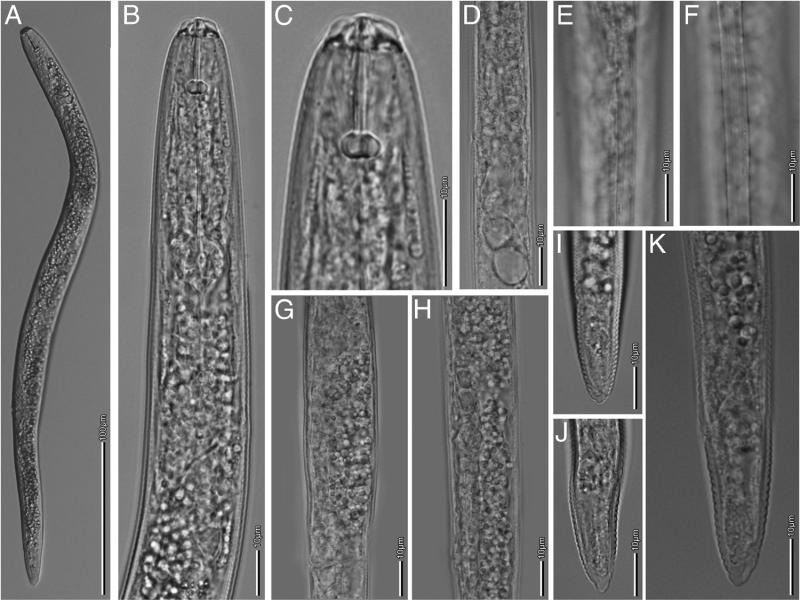
Light micrographs of female *Pratylenchus zeae*. A: Entire body; B: Anterior region; C: Head; D: esophageal region; E-F: Lateral field at mid body; G-H: Reproductive tract showing small round spermatheca; I-K: Tail; (scale bars: B-K, = 10 μm; A = 100 μm).

***Pratylenchus zeae*** Graham, 1951.

(Figure 5 and Table 5).

**Table 5.** Measurements of two *Pratylenchus zeae* populations from Ghana and Nigeria.

Sample	Umuagu 1	Kintampo S 1	
n	1♀	2♀♀*	4♀♀
L	382	381–561	433±36.9 (403–483)
a	23.1	17.5–17.4	19.9±2.7 (17.1–23.5)
b		–	4.5±0.36 (4.0–4.8)
b’		–	4.5±1.3 (3.2–6.3)
c	28.7	15.6–21.7	17.6±2.1 (14.7–19.7)
c’	1.8	1.8–1.7	2.0±0.24 (1.6–2.1)
V%	70.3	70.0–70.8	71.8±1.1 (70.7–73.2)
Stylet length	15.7	14.6–14.7	15.9±0.95 (14.6–16.9)
Stylet knob width		4.9–4.7	4.1±0.4 (3.7–4.4)
Stylet knob height		2.9–3.3	2.4±0.29 (2.1–2.7)
DEGO from stylet base	3.5	–	2.9±0.21 (2.6–3.1)
*Anterior end to:*			
centre of metacorpus	44.1	47.4–	50.3±3.1 (47.3–53.7)
median bulb base		55.9–	57.6±3.3 (53.9–60.7)
cardia		–	95.7±8.3 (84.2–103)
end of esophageal gland end	116	–	101±24.1 (69.6–126)
secretory/excretory pore	69.1	72.0–	71.1±7.9 (64.8–82.6)
Esophagus overlap		21.4–23.9	22.3±4.3 (17.1–26.5)
Max. body diam.	16.5	21.8–32.3	21.9±1.4 (20.5–23.8)
Vulval body diam.	15.3	20.8–24.2	19.6±1.2 (18.3–20.7)
Anal body diam.	7.2	13.3–15.0	12.9±0.89 (12.0–14.1)
Anterior genital		–	159±27.5 (121–183)
Spermatheca-vagina		–	36.3±3.5 (31.6–40.2)
Tail length	13.3	24.5–25.9	24.8±3.2 (20.4–27.7)
Number of tail annuli		–	19.3±1.5 (18.0–21.0)
Vulva to anus distance	110	93.2–149	94.2±7.4 (85.6–103)
Post-uterine sac	25.8	19.6–30.4	21.5±5.3 (15.3–26.5)
Lateral field width		–	7.9±1.2 (6.5–9.3)

* Morphometrics derived from temporary slides; otherwise, morphometrics derived from permanent slides. All measurements are in μm and in the form: mean ± s.d. (range).

Female:

Body slender, short 381–561 μm long, and near-straight when heat-relaxed. Cephalic region continuous with body and bearing three annuli. Lateral fields with four lines at mid body. Stylet 14.6–16.9 µm long, with broad, anteriorly flattened basal knobs. Esophageal glands overlapping intestine ventrally and laterally. Ovary usually long. DEGO at 3 μm posterior to the stylet base. Excretory pore just anterior to the esophago-intestinal junction. Spermatheca rounded, without sperm. Vulva at 70–73.2%. Post-vulval uterine sac short, about 1 body diam. long. Tail tapering, with 18–21 annuli terminating in an almost pointed tip.

Males: Not observed.

Remarks: Based on the morphology and the morphometrics, the studied populations were in agreement with the original description of *P. zeae* and to the neotype female and other descriptions of *P. zeae* (Fortuner, 1976; Castillo and Vovlas, 2007).

### Molecular characterization of *Pratylenchus* spp. from yam

#### The D2-D3 of 28 S rDNA gene

**Figure 6:**
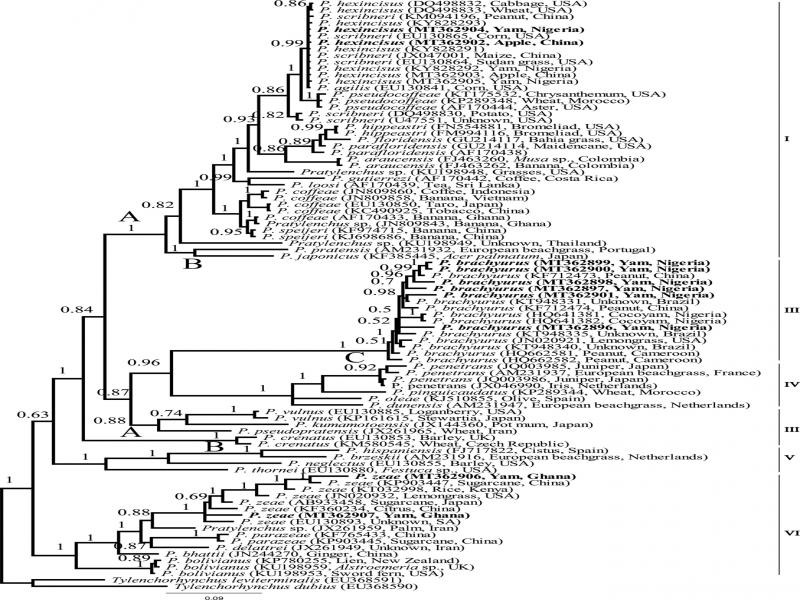
Bayesian 50% majority rule consensus tree from four runs as inferred from analysis of the D2-D3 of 28 S rRNA gene sequence alignment under the GTR + I + G model. (-lnL = 11091.5259; AIC = 22563.051780; freqA = 0.1873; freqC = 0.2354; freqG = 0.3250; freqT = 0.2523; R(a) = 1.0893; R(b) = 3.9431; R(c) = 2.1703; R(d) = 0.4799; R(e) = 5.3436; R(f) = 1.0000; p-inv = 0.3210; gamma shape = 0.8480). Posterior probability values exceeding 50% are given on appropriate clades). New sequences are indicated by bold font.

The D2-D3 alignment included 80 *Pratylenchus* sequences, and two outgroup sequences. Thirteen new D2-D3 sequences were obtained in the present study. Following the numbering proposed by Subbotin et al. (2008), the BI tree contained five highly supported clades except for clade III (Figure 6).

The sequences of *P. hexincisus* generated in this study formed a very well supported clade without internal resolution with *P. hexincisus* sequences from China (MT362902 and MT362903), *P. hexincisus* sensu Inserra et al., 2007 obtained from the type locality (DQ498832 and DQ498833), *P. scribneri* Steiner in Sherbakoff & Stanley, 1943 (EU130864, EU130865, JX047001 and KM094196) and *P. agilis* (Thorne and Malek, 1968) (EU130841). However, sequences of *P. scribneri sensu* Inserra et al. (2007) (DQ498830) and *P. scribneri* from California U47554 (Al-Banna et al., 1997) formed a separate clade. The intraspecific variation of our *P. hexincisus* populations was 1–2 bp (0.1–0.3%) and differed only 0–2 bp (0–0.3%) with *P. hexincisus* from the type location (Inserra et al., 2007) (DQ498832 and DQ498833) and 0–3 bp (0–0.4%) with *P. agilis* (EU130841) and 1–5 bp (0.1–0.6%) with *P. scribneri* (EU130864, EU130865, JX047001 and KM094196), while it was clearly different (14–17 bp, 2.5–5.7%) from *P. scribneri sensu* Inserra et al. (2007) (DQ498830).

Sequences of *P. brachyurus* from this study, together with *P. brachyurus* sequences from GenBank were grouped in a well-supported subclade C of the clade III. The intraspecific variation of *P. brachyurus* was 2–51 bp (0.3–6.6%) and nucleotide difference between *P. brachyurus* and the most similar sequence, *P. penetrans,* was 152–177 bp (19.5–23%).

*Pratylenchus zeae* sequences formed a well-supported clade together with *P. zeae* sequences from GenBank. The intraspecific sequence variation of *P. zeae* was 23–65 bp (3.2–9%) and the interspecific sequence difference with the closest related species, *Pratylenchus* sp. (JX261959), was 23–80 bp (3.2–11.1%).

#### The mitochondrial *COI* gene

**Figure 7:**
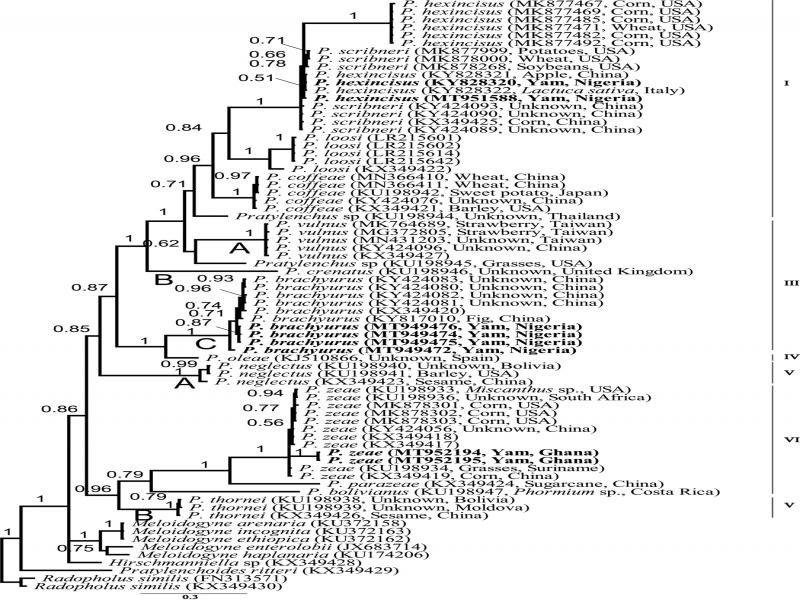
Bayesian 50% majority rule consensus tree from four runs as inferred from analysis of the *COI* mtDNA gene sequence alignment under the GTR + I + G model. (-lnL = 6250.9879; AIC = 12821.975780; freqA = 0.2952; freqC = 0.0915; freqG = 0.1808; freqT = 0.4325; R(a) = 0.1894; R(b) = 6.2295; R(c) = 1.8356; R(d) = 5.2302; R(e) = 5.3626; R(f) = 1.0000; p-inv = 0.2340; gamma shape = 0.6370). Posterior probability values exceeding 50% are given on appropriate clades). Original sequences are indicated by bold font.

The *COI* sequences alignment was 422 bp in length and included 58 sequences of *Pratylenchus* including eight newly generated sequences, and four outgroup taxa (*Meloidogyne*, *Hirschmanniella*, *Pratylenchoides* and *Radopholus*). The BI tree contained five highly supported clades following numbering proposed by Subbotin et al. (2008) (Figure 7).

Sequences of *P. hexincisus* from yam formed a well-supported clade with *P. hexincisus* sequences from China, Italy and the USA and *P. scribneri* sequences from China and the USA, with *P. loosi* (PP 0.84) as sister species. The sequences of *P. hexincisus* generated in this study and *P. hexincisus* sequences from Italy (KY828322) and China (KY828321) and *P. scribneri* (MK877999: USA; MK878000: USA; MK878268: USA, KY424093: China; KY424090: China; KY424089: China; KX349425: China) were very similar 0–8 bp (0–1.93%). However, these sequences were different from the recently deposited *P. hexincisus* sequences from Wheat and Corn in the USA (MK877467, MK877469, MK877471, MK877482, MK877492) with 51–81 bp (19.8–21.1%). Sequences of the closest related species, *P. loosi,* differed 54–102 bp (19.1–24.5%).

Sequences of *P. brachyurus* from this study, together with other *P. brachyurus* sequences available in the NCBI GenBank database formed a well-supported subclade C of clade III, sister to *P. oleae* (clade IV) (Palomares-Rius et al., 2014). The intraspecific variation of *P. brachyurus* was 0–16 bp (0–4.1%) and the interspecific sequence difference between *P. brachyurus* and *P. oleae* was 78–81 bp (21.1–22%).

*Pratylenchus zeae* sequences formed a well-supported clade (VI) together with *P. zeae* sequences from GenBank. The intraspecific sequence variations of *P. zeae* were 0–37 bp (0–9.6%) and the interspecific sequence difference was 99–112 bp (25.9–28.6%) with *P. parazeae*, the closest related species.

## Discussion

Prior to the current study, seven RLN species, i.e. *P. brachyurus*, *P. crenatus* (Loof, 1960), *P. coffeae, P. loosi* (Loof, 1960)*, P. sudanensis*, *P. pseudopratensis* and *P. zeae* have been reported from yam rhizosphere and yam tubers (Caveness, 1967; Bridge, 1973, 1988; Coyne et al., 2003; Varghese and Mohandas, 2004; Bridge and Starr, 2007; Mudiope et al., 2007; Osei et al., 2015; Coyne et al., 2018). Using a combination of morphological and molecular identification, *P. brachyurus* and *P. hexincisus* were identified from yam tubers, while *P. zeae* was recovered from the yam rhizosphere only. *Pratylenchus brachyurus,* a cosmopolitan species, appears as the predominant species on yam in Nigeria and Ghana, which is in agreement with other studies that have reported *P. brachyurus* from yam in Nigeria and West Africa (Luc and de Guiran, 1960; Unny and Jerath, 1965; Caveness, 1967; Bridge, 1972, 1973). In this region, the polyphagous *P. brachyurus* has also been recorded as a pest of numerous crops (Miège, 1957; Luc and de Guiran, 1960; Bridge, 1973; Egunjobi, 1974; Egunjobi and Larinde, 1975; Guerout, 1975; Coyne et al., 1999; Castillo and Vovlas, 2007), including an interception from *Colocasia* sp. (another tuber crop) from Nigeria to China (Zhao et al., 2011). Also, it is known to affect plant growth and the yield of crops in West Africa, for instance on pineapple (Guerout, 1975) and cassava (De Guiran, 1965).

*Pratylenchus* species, and in particular *P. coffeae* are known to cause “dry rot” on yam tubers, a condition similar to that caused by *S. bradys,* based on what is known for *P. coffeae* and *P. sudanensis* (Bridge et al., 2005; Bridge and Starr, 2007; Coyne and Affokpon, 2018). However, symptoms of *P. brachyurus* or its effects on yam production are not well known, Given the predominance of *P. brachyurus* in yam tubers and yam rhizosphere, it appears that this species is a major RLN on yam in West Africa. However, more work is necessary to clearly establish the effect of this species on yam growth, yield and tuber quality. The ability of *P. brachyurus* to survive a long period without a host and its polyphagous nature, could make its management particularly difficult, without the use of resistant cultivars.

*Pratylenchus zeae*, retrieved only from the yam rhizosphere of one sample in Ghana and one in Nigeria, is a commonly occurring species on other crops in West Africa (Fortuner, 1976; Plowright and Hunt, 1994; Coyne et al., 1996; Castillo and Vovlas, 2007). *Pratylenchus zeae* was reported on yam in Nigeria (Bridge, 1973) but has never been reported on yam rhizosphere in Ghana. Its absence from tuber tissue, however, indicates that yam tubers may not support *P. zeae* and that its occurrence in this case may be related to other plant species occurring together with the sampled yam.

*Pratylenchus coffeae*, one of the major plant-parasitic nematodes of yam in the Americas and the Pacific Islands was not recorded in any of the samples collected from Ghana and Nigeria. A similar observation was reported by Kwoseh et al. (2005) in Ghana. This remarkable absence from *P. coffeae* supports the statement of Duncan and Moens (2013) that “*P. coffeae* is a pest of yam, interestingly, not in Africa”, despite being present on other crops in both localities (Duncan et al., 1999; Pourjam et al., 1999; Speijer et al., 2001; Bridge et al., 2005; Kwoseh et al., 2005; Coyne and Affokpon, 2018). Although Osei et al. (2015) recorded *P. coffeae* on yam in Ghana, but its identity was not ascertained by molecular method.

Traditional taxonomy can have serious limitations for differentiating species of *Pratylenchus* (Luc, 1987; Subbotin et al., 2008). In the current study, however, populations of *P. brachyurus* were, despite a remarkable intraspecific variation, relatively easily identified based on morphology and morphometrics, including the number of lip annuli (2), stylet length (17–21 µm), vulva position (77–88%), and a bluntly rounded tail, which while highly variable was never conically pointing, posteriorly confirmed by molecular data. Our observations agree with the descriptions provided by Corbett (1976) and Castillo and Vovlas (2007), including its well-known intraspecific variation on the tail, lips and knobs shape (Roman and Hirschmann, 1969; Corbett, 1976; Tarjan and Frederick, 1978; Payan, 1989). However, the presence of a developed sperm-filled spermatheca in 2 of the 108 analysed specimens was observed for the first time.

In this study, *P. hexincisus* was recorded from yam for the first time, although just from one sample. The relatively high number of specimens retrieved from yam peels unequivocally demonstrates its association with the tuber. Therefore, infection studies to prove Koch’s postulates are required to demonstrate that *P. hexincisus* is a pest of yam. *Pratylenchus hexincisus* recorded from Benue State, Nigeria, is morphologically very similar to *P. scribneri,* which has been reported on maize in the neighbouring Western region of Nigeria (Anonymous, 1975). Molecular researches are necessary to establish if both species represent two different species or are conspecific.

The observed morphology and morphometrics of *P. hexincisus* agreed with the original description (Taylor and Jenkins, 1957), although variability in the number of lines in the lateral field, with four to six lines have been observed. This variation is known for *P. brachyurus* (Castillo and Vovlas, 2007) as well as other members of the genus *Pratylenchus*, for example four to seven lines in the lateral field have been reported in *P. neglectus* (Rensch, 1924) Filipjev and Schuurmans Stekhoven, 1941 (Corbett and Clark, 1983) and four to six lines in *P. scribneri* (Roman and Hirschmann, 1969; Loof, 1985; Inserra et al., 2007). Originally described from corn in Maryland, USA (Taylor and Jenkins, 1957), *P. hexincisus* was distinguished as a new species, separate from *P. scribneri*, by the presence of 6 lines in the lateral field, its smaller size and the fact that no spermatheca was observed. However, morphological studies of both species have revealed high morphological similarities, including the presence of empty spermatheca and variation in the number of lines in the lateral field (Roman and Hirschmann, 1969; Corbett and Clark, 1983; Castillo and Vovlas, 2007; Inserra et al., 2007; Ozbayrak et al., 2019). Yet, a comprehensive investigation by Inserra et al., (2007), including *P. hexincisus* from the type locality (DQ498832-33) and a reference population of *P. scribneri* (DQ498830), showed a molecular distinction between *P. hexincisus* and *P. scribneri,* based on the D2-D3 region of the 28 S rDNA. Moreover, this reference *P. scribneri* population (DQ498830) also formed a distinct clade with *P. scribneri* (U47551) reported by Al-Banna et al. (1997). Furthermore, the authors also highlighted morphological characters that could discriminate both species, including the presence of a rectangular-elongated spermatheca in *P. hexincisus* versus rounded in *P. scribneri*. However, although the D2-D3 sequences of the isolates of yam were virtually identical to the sequences of *P. hexincisus* (Inserra et al., 2007), our population did not show an elongated spermatheca but always a rounded spermatheca. On the other hand, our population showed crenated outer incisures of the lateral field (Figure 4) as described in the original description of *P. hexincisus* (Taylor and Jenkins, 1957). The number of lateral lines appeared to be less discriminative to distinguish *P. hexincisus* and *P. scribneri* as six lateral lines were also observed in *P. scribneri* (Inserra et al., 2007). Similar variability on the lateral lines was observed in *P. brachyurus* reported in this study indicating that caution is needed when using this character to discriminate species in the genus *Pratylenchus*.

Although the D2-D3 sequences of *P. hexincisus* in our study were similar with those of *P. scribneri* from Imperial Valley, California and Vero Beach, Florida (EU130864-65) the uncertainty of the identification of the isolates was already mentioned by Subbotin et al. (2008) and these sequences may therefore represent *P. hexincisus*.

Augmenting the problem to delimitate both species, the COI sequences *P. hexincisus* (MK877467, MK877469, MK877485, MK877471, MK877482, MK877492) in the study reported by Ozbayrak et al. (2019) were clearly different from *P. hexincisus* from yam when assessed in the current study. Remarkably, our *P. hexincisus* COI sequences were similar to the sequences of *P. scribneri* (MK877999, MK878000, MK878268) Ozbayrak et al. (2019). Also, *P. scribneri* D2-D3 and COI sequences from China (JX047001, KM094196, KX349425) were similar to our *P. hexincisus* sequences and these Chinese D2-D3 sequences are also similar to those from the *P. hexincisus* type locality (Inserra et al., 2007). The unclear identity of the COI sequences could be resolved if the materials from Al-Banna et al. (1997) and Inserra et al. (2007) could be linked to COI sequences, i. e. if the *P. hexincisus* and *P. scribneri* COI sequences *sensu* Ozbayrak et al. (2019) agree with the identity of the material from Al-Banna et al. (1997) and Inserra et al. (2007). However, this is likely not the case since, according to the supplementary D2D3 tree in Ozbayrak et al. (2019), their *P. scribneri* sequences are different to the *P. scribneri* (DQ498830) from Inserra et al. (2007).

In summary, *P. hexincisus* and *P. scribneri* have similar, indeed overlapping, morphometric characteristics and shared morphological characters, leading to a confuse and difficult identification. Hence, a topotype population of *P. scribneri* is needed to solve the identity and validity of *P. scribneri* and *P. hexincisus,* as suggested by Inserra et al. (2007) and Subbotin et al. (2008). Likewise, the D2-D3 sequence of *P. agilis* Thorne and Malek, 1968 is also similar to *P. hexincisus sensu* Inserra et al. (2007), as provided and mentioned by Subbotin et al. (2008). Loof (1978) had already doubted the validity of *P. agilis* and the species was considered as *species inquerendae* (Frederick and Tarjan, 1989). This was confirmed by ITS sequences and isozyme analysis. *Pratylenchus agilis* was proposed as a junior synonym of *P. scribneri* (Hernández et al., 2000), although Waeyenberge et al. (2000) indicated differences between *P. scribneri* and *P. agilis* with respect to ITS-rDNA length and the RFLPs.

Evidently, additional morphological and molecular characterizations are required to further analyses the species group of *P. scribneri*, *P. hexincisus* and *P. agilis*.
